# BZR1, you have an invite: EPFL–ERECTA wants to join your female germline specification network

**DOI:** 10.1093/plcell/koad045

**Published:** 2023-02-17

**Authors:** Jessica Franco

**Affiliations:** Assistant Features Editor, The Plant Cell, American Society of Plant Biologists, USA; Department of Plant Pathology, Washington State University, Pullman, Washington, USA

Plant reproduction relies on the effective development of the female gametophyte. The megaspore mother cell (MMC) marks the first cell of the plant female germline. The MMC undergoes meiosis and mitosis to form the mature female gametophyte. Exactly how MMC formation is restricted to a single cell remains unknown. However, multiple signaling complexes are involved in MMC formation. Particularly, the brassinosteroid (BR) signaling module BRI1–BZR1 upregulates the WRKY23 transcription factor to prevent the surrounding sub-epidermal cells from acquiring MMC identity (Cai et al. 2022). Similarly, loss-of-function mutants in the ERECTA receptor-like kinase family, (ERf), result in MMC formation defects (Hou et al. 2021). In this issue of *The Plant Cell*, **Hanyang Cai and colleagues (**Cai et al. 2023**)** identified that BRI1–BZR1 and the ERf signaling pathways interconnect to control MMC formation and specification in Arabidopsis (see Figure).

**Figure.**
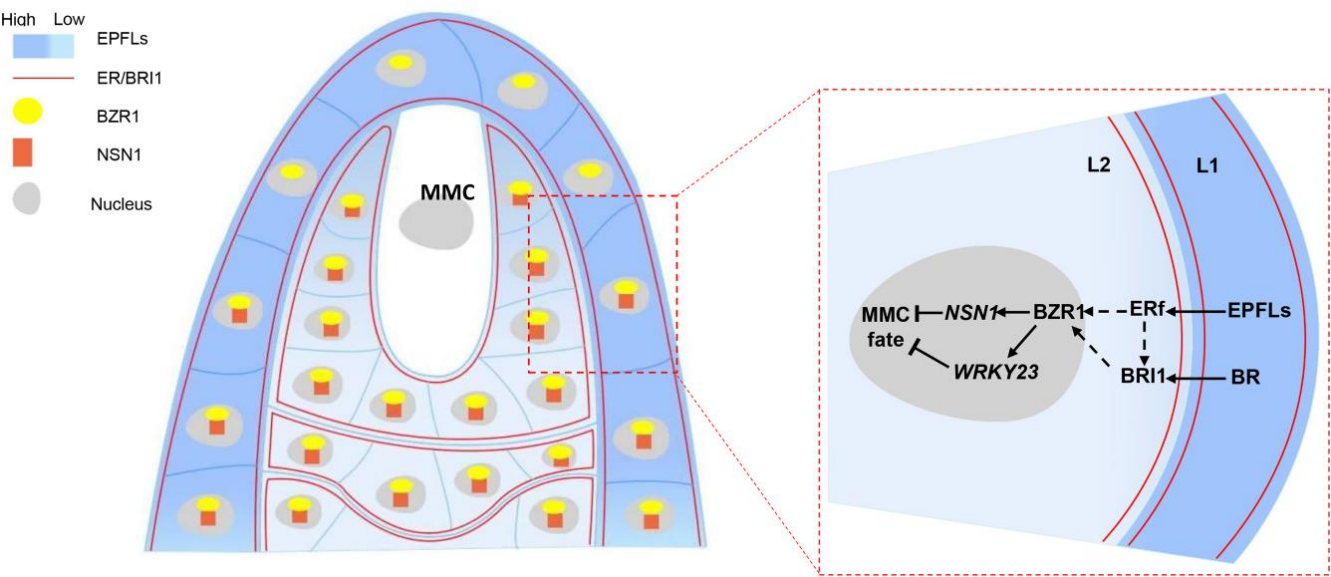
Model of EPFL-ERf-BZR1 signaling pathway regulating MMC specification. In Arabidopsis, EPFL peptides are perceived by the ERECTA family (ERf) in the ovule primordium. The EPFL–ERf complex activates the BRI1–BZR1 signaling pathway. BZR1 transcription factors regulate NSN1 and WRKY23 to suppress the surrounding sub-epidermal cells from gaining MMC identity. Reprinted from Cai et al. (2023), Figure 7.

ERfs (ERECTA, ERECTA-like 1 (ERL1) and ERL2) perceive the EPIDERMAL PATTERNING FACTOR (EPF)/EPF-like (EPFL) family of conserved cysteine-rich peptides. The current model suggests EPFLs are ligands during female fertility. The authors tested the spatial–temporal expression of *EPFL*s and *ERf*s using transcriptional reporter assays. The *EPFL*s, *EPFL1*, *EPFL2*, *EPFL4,* and *EPFL6* co-expressed with the *ERf* members within the ovule primordia. Phenotypic analyses of single, double, triple, and quadruple *EPFL* mutants showed that *EPFL*s function additively in fertility.

To link ERfs and EPFLs to MMC formation, the authors immunostained the ovules of the triple ERf mutant, *er erl1 erl2*, and quadruple EPFL mutant, *epfl1,2,4,6*. MMC markers localized to multiple enlarged cells that were observed in both mutants, indicating that multiple cells acquired MMC identity. However, only one MMC-like cell in the mutant lines could undergo meiosis. These results suggest that the EPFL–ERf ligand–receptor complex restricts MMC identity to a single cell, but other factors are required to restrict meiosis to a single MMC cell.

To connect EPFL–ERf and BR signaling pathways, the authors tested *BRI1* and *BZR1* gene expression in the *epfl1,2,4,6* and *er erl1 erl2* higher order mutants. *BRI1* and *BZR1* expression was significantly reduced in both lines. As such, the authors hypothesized that the BRI1–BZR1 pathway functions downstream of the EPFL–ERf complex. After BR treatment, BZR1 is activated via dephosphorylation (Chen et al. 2019). Therefore, the authors visualized BZR1 phosphorylation after EPFL peptide treatment via immunoblotting in wild-type Arabidopsis plants. In comparison to a mock treatment, activated BZR1 accumulated at higher levels confirming that BRI1–BZR1 is activated by the ERf–EPFL pathway.

The BZR1 transcription factor family regulates over 7,000 genes (Sun et al. 2010). Among those is a nucleolar GTP-binding protein, *Nucleostemin*-*like* 1 (*NSN1*), that is preferentially expressed in the pineapple ovule during the MMC stage (Zhao et al. 2021). To validate BZR1-mediated *NSN1* expression, the authors performed chromatin immunoprecipitation and electrophoretic mobility shift assays, which showed that BZR1 binds to the E-boxes in the *NSN1* promoter region. The BZR1–*NSN1* promoter binding is dependent upon EPFL1 treatment. These results support the conclusion that EPFL–ERf signaling activates BZR1 to regulate *NSN1* expression.

To test whether *NSN1* is the downstream factor for MMC formation, the authors ectopically expressed *NSN1* in the ovules of the BZR1 family quintuple mutant that also exhibits multiple MMC-like cells (Cai et al. 2022). *NSN1* expression partially complemented the MMC-like phenotype suggesting that NSN1 is not the sole molecular player. Since it was known that BZR1 also upregulates *WRKY23* (Cai et al. 2022), the authors generated *nsn1 wrky23* double mutants. Multiple MMC-like cells were observed at a significantly higher frequency than wild-type or *nsn1* and *wrky23* single mutants, and immunostaining confirmed that the cells possess MMC-like characteristics. These results illustrate that both *NSN1* and *WRKY23* function in MMC-like specification.

This study provided new insights into the components that control and restrict the initiation of female germline development and introduces EPFL–ERECTA as a member of the female germline specification network. Further insights into additional factors controlling female germline initiation and specification can lead to useful breeding tools.
